# Infant Respiratory Syncytial Virus Immunization Through Maternal Vaccination and Nirsevimab

**DOI:** 10.1001/jamanetworkopen.2025.59663

**Published:** 2026-02-16

**Authors:** Karen P. Acker, Kevin Strobino, Jessica M. DeAngelis, Anna P. Staniczenko, Moeun Son, Laura E. Riley, Jin-Young Han, Erika L. Abramson, Zachary M. Grinspan, Deborah A. Levine

**Affiliations:** 1 Department of Pediatrics, Weill Cornell Medicine, New York, New York; 2 Department of Medicine, Weill Cornell Medicine, New York, New York; 3 Department of Obstetrics and Gynecology, Weill Cornell Medicine, New York, New York; 4 Department of Population Health Sciences, Weill Cornell Medicine, New York, New York; 5 Department of Emergency Medicine, Weill Cornell Medicine, New York, New York

## Abstract

**Importance:**

In 2023, 2 forms of respiratory syncytial virus (RSV) prevention, maternal RSV vaccine and nirsevimab, became available for infants. The factors that played a role in their uptake during the first 2 seasons remain unclear.

**Objective:**

To describe the rate of RSV immunization through maternal RSV vaccination or nirsevimab during the 2023-2024 and 2024-2025 RSV seasons and identify the factors associated with their receipt.

**Design, Setting, and Participants:**

This cohort study was performed at clinical sites affiliated with a quaternary care hospital in New York, New York, from October 1, 2023, to March 31, 2024, and October 1, 2024, to March 31, 2025. Participants included infants younger than 8 months who had at least 1 clinical encounter during the 2023-2024 and 2024-2025 RSV seasons.

**Main Outcomes and Measures:**

The primary outcomes were receipt of RSV immunization by maternal RSV vaccine 14 days or more prior to delivery or nirsevimab. No evidence of RSV immunization was defined as either no documentation of either product or receipt of maternal vaccine less than 14 days prior to delivery without subsequent nirsevimab immunization. A multinomial logistic regression model was used to estimate odds ratios (ORs) controlling for age, sex, RSV season, insurance, and race and ethnicity, to assess factors associated with vaccination.

**Results:**

Of 13 195 eligible infants (6831 [51.8%] male; 8367 [63.4%] newborn; median age of nonnewborn infants, 17.7 [IQR, 8.1-26.9] weeks), 11 804 of 12 913 (91.4%) were born at term (gestational age ≥37 weeks), 11 208 of 12 964 (86.5%) were privately insured, and 12 109 of 13 195 (91.8%) were born at the study institution. A total of 8830 infants (66.9%) received RSV immunization through maternal RSV vaccine (3832 [29.0%]) or nirsevimab (4998 [37.9%]). RSV immunization coverage increased from 3595 of 6245 infants (57.6%; maternal RSV vaccine, 1317 [21.1%]; nirsevimab, 2278 [36.5%]) in the 2023-2024 season to 5235 of 6950 (75.3%; maternal RSV vaccine, 2515 [36.2%]; nirsevimab, 2720 [39.1%]) in the 2024-2025 season. Increased odds of receiving maternal RSV vaccine (adjusted OR [AOR], 3.58; 95% CI, 3.22-3.99) and nirsevimab (AOR, 1.89; 95% CI, 1.73-2.06) were associated with the 2024-2025 season compared with the 2023-2024 season. Lower odds of receiving maternal RSV vaccine (AOR, 0.18; 95% CI, 0.15-0.22) or nirsevimab (AOR, 0.80; 95% CI, 0.70-0.89) were associated with public compared with private insurance.

**Conclusions and Relevance:**

In this cohort study of eligible infants younger than 8 months, receipt of RSV immunization through maternal RSV vaccine or nirsevimab improved in the 2024-2025 compared with the 2023-2024 RSV seasons. However, infants with public insurance were less likely to receive either product, highlighting persistent disparities in RSV immunization warranting targeted interventions.

## Key Points

**Question:**

What factors were associated with receipt of respiratory syncytial virus (RSV) immunization by maternal RSV vaccine or nirsevimab in infants during the 2023-2024 and 2024-2025 RSV seasons?

**Findings:**

In this cohort study of 13 195 infants who were seen for medical care before 8 months of age, 58% of 6245 infants and 75% of 6950 infants received maternal RSV vaccine or nirsevimab in the 2023-2024 and 2024-2025 RSV seasons, respectively. Infants with public insurance had lower odds of receiving either type of RSV immunization.

**Meaning:**

These findings suggest that despite improved RSV immunization, socioeconomic differences affected receipt of RSV immunization in infants.

## Teaser

This cohort study assesses receipt of maternal respiratory syncytial virus vaccine or infant nirsevimab immunization in a large cohort of infants in a major urban medical system during the first 2 seasons these products were available.

## Introduction

Respiratory syncytial virus (RSV) causes more than 50 000 hospitalizations and as many as 300 deaths per year in children younger than 5 years in the US.^1,2,3^ Two products targeted to RSV prevention in infants, maternal RSV vaccine and the monoclonal antibody nirsevimab, were approved by the US Food and Drug Administration and recommended by the Advisory Community on Immunization Practices prior to the 2023-2024 RSV season.^4,5^ Infants can receive RSV immunization either through maternal-fetal antibody transfer from vaccination of the pregnant mother with the nonadjuvanted bivalent RSV prefusion F protein–based vaccine (Pfizer Inc) administered between 32 and 36 weeks of pregnancy at least 14 days prior to delivery^6^ or through administration of the long-lasting monoclonal antibody nirsevimab to infants younger than 8 months entering their first RSV season or children aged 8 to 19 months with increased risk for severe RSV disease entering their second RSV season.^5,7^

The initial rollout of maternal RSV vaccine and nirsevimab in 2023 to 2024 was hindered by multiple challenges. Most notably, the pregnancy vaccination recommendations were released weeks into RSV season,^4^ and there was a limited national supply of nirsevimab, which led the Centers for Disease Control and Prevention (CDC) to recommend restrictions on eligibility.^8^ Single-center reports of the initial experience with RSV immunization demonstrated heterogeneity in uptake of maternal RSV vaccine and nirsevimab immunization due to variable supply, limited public awareness, insurance reimbursement issues, and inconsistent offerings across clinical settings.^9,10,11,12,13^ Additionally, disparities were apparent in the initial rollout, with lack of immunization receipt associated with being a member of a racial and ethnic minority group and having public insurance.^10,11,12,14,15^ The factors associated with the uptake of maternal RSV vaccine and nirsevimab in the second RSV season (2024-2025) have, to our knowledge, not yet been evaluated.

We sought to determine the prevalence of maternal RSV vaccination or infant nirsevimab immunization in a large cohort of infants in a major urban medical system during the first 2 RSV seasons (2023-2024 and 2024-2025) these products were available. We also sought to determine demographic and clinical factors associated with receipt of RSV immunization.

## Methods

### Study Design and Population

This study was approved by the Weill Cornell Medicine Institutional Review Board with a waiver of informed consent because the study involved only minimal risk using data collected during routine medical care, obtaining consent would be impracticable due to the large sample size and multiple sites, and the waiver would not adversely affect subjects’ rights or welfare. Our findings follow the Strengthening the Reporting of Observational Studies in Epidemiology (STROBE) reporting guideline.

A retrospective observational cohort study was performed during the first 2 RSV seasons after maternal RSV and nirsevimab became available in a quaternary care hospital in New York, New York. The study population included all infants who were younger than 8 months from October 1 to March 31 in both the 2023-2024 and 2024-2025 seasons and had an encounter at a clinical site affiliated with Weill Cornell Medicine, including the newborn nurseries, neonatal intensive care unit, outpatient clinics, inpatient pediatric units, and emergency departments. Infants who did not have vaccination records available were excluded.

Eligible patients were identified in our electronic health record (EHR) system, and infant and maternal immunization data were extracted using direct structured language queries. Vaccination records for both maternal RSV vaccine and nirsevimab were captured if administered at any affiliated clinical site, imported through Epic Care Everywhere (Surety Systems) from outside health systems, or obtained from city and state immunization registries (New York Citywide Immunization Registry and New York State Immunization Information System), which supply all childhood immunization data. Maternal vaccinations were additionally captured if administered at a pharmacy owned by a pharmacy benefit manager, electronically prescribed to a pharmacy not owned by a pharmacy benefit manager and reconciled by the pharmacy, or manually entered based on patient report. Maternal vaccination status was determined by linking birth and infant records in Epic STORK (Epic Systems) or through maternal contact information in the EHR. When no linked maternal record was found, maternal RSV vaccination was classified as mother not documented.

Additional variables collected included age, gestational age, sex, self-reported race and ethnicity, insurance used at visit, comorbidities, and preferred language. Study age was defined according to RSV immunization status as age at nirsevimab receipt (0 for infants with maternal immunization) and age at last clinical encounter during the RSV season for infants without documentation of RSV immunization. Newborns were defined as study age less than 7 days. Race and ethnicity were extracted from self-reported demographic fields in the EHR, where they were captured as separate variables. For analysis, race and ethnicity were combined into mutually exclusive categories: Hispanic, non-Hispanic Asian, non-Hispanic Black, non-Hispanic White, and non-Hispanic other (including American Indian or Alaska Nation, Native Hawaiian or Pacific Islander, and other combinations not described). Race and ethnicity were collected as prior studies have demonstrated disproportionate receipt of RSV immunization among different racial and ethnic groups.^10,11,12,14,15^ Comorbidities were estimated with the Pediatric Medical Complexity Algorithm for complex chronic disease.^16^ Clinical location was defined as the location of nirsevimab receipt or the last location an infant was seen during the RSV season.

### Definitions of RSV Immunization

Maternal RSV immunization was defined as documented maternal vaccination at least 14 days prior to delivery. Nirsevimab immunization was defined as documented receipt by an infant younger than 8 months. Infants were classified as having no evidence of RSV immunization if they had neither documented maternal vaccination nor nirsevimab or if maternal vaccination occurred less than 14 days before delivery without subsequent nirsevimab immunization.

### Statistical Analysis

Descriptive statistics performed included median of continuous variables and number and percentage of categorical variables. To evaluate which factors were associated with RSV immunization status, a multinomial logistic regression model was used and outcomes included maternal RSV vaccine receipt, nirsevimab receipt, or no evidence of RSV immunization (reference category). The factors included were newborn status (age <7 vs ≥7 days), sex, RSV season, race and ethnicity (Hispanic, non-Hispanic Asian, non-Hispanic Black, or non-Hispanic White), insurance status (public vs private), and preterm status (gestational age <37 vs ≥37 weeks). To account for the recommended timing of maternal RSV vaccination, a dummy variable was included to account for the time when maternal RSV is recommended (September to January) or not recommended (February to March). Complete case analysis was performed. To enable direct comparison between receipt of maternal RSV vaccine and nirsevimab, we also respecified the model using nirsevimab as a reference category. Multiple sensitivity analyses were performed to account for infants excluded for missing vaccine records and with overlapping receipt of both RSV products (eMethods in Supplement 1). Adjusted odds ratios (AORs) and 95% CIs were reported. Pearson χ^2^ test was used to compare the distribution of clinical locations where nirsevimab was received between RSV seasons. To evaluate whether observed seasonal differences in the clinical location of nirsevimab administration could be explained by patient characteristics, we conducted a secondary analysis in which clinical location was included in the model as an interaction term with season (eMethods in Supplement 1). All statistical analyses used a significance threshold of 2-sided α = .05 and were performed using SAS software, version 9.4 (SAS Institute Inc).

## Results

### Study Population

**Figure 1.**
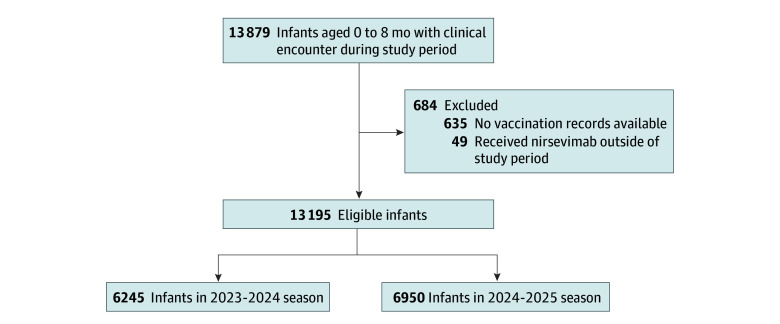
Flow Diagram of Study Population

From October 1 to March 31 of the 2023-2024 and 2024-2025 RSV seasons, 13 195 eligible infants were identified after excluding 635 with unavailable vaccination records and 49 who received nirsevimab outside the study period, 6245 (47.3%) from the 2023-2024 and 6950 (52.7%) from the 2024-2025 RSV seasons (Figure 1). A total of 6363 infants (48.2%) were female, 6831 (51.8%) were male, and 1 (0.01%) unknown; 11 804 of 12 913 (91.4%) were born at term (≥37 weeks gestation); 11 208 of 12 964 (86.5%) were privately insured; and 12 109 of 13 195 (91.8%) were born at the study institution. For race and ethnicity, 1930 (14.6%) were Hispanic; 2270 (17.2%), non-Hispanic Asian; 756 (5.7%), non-Hispanic Black; 7085 (53.7%), non-Hispanic White; 319 (2.4%), non-Hispanic other; and 835 (6.3%), unknown (Figure 1 and Table 1). Most infants were newborns (8367 [63.4%]), and the median age of nonnewborns was 17.7 (IQR, 8.1-26.9) weeks.

**Table 1.** Characteristics of Infants Aged 0 to 8 Months Eligible for RSV Immunization Through Maternal RSV Vaccination or Nirsevimab

Characteristic	Patient group, No. (%)				
	Overall (N = 13 195)	No evidence of RSV immunization (n = 4365 [33.1%])	RSV immunization (n = 8830 [66.9%])	Maternal RSV vaccine (n = 3832 [29.0%])	Nirsevimab (n = 4998 [37.9%])
RSV season					
2023-2024	6245 (47.3)	2650 (60.7)	3595 (40.7)	1317 (34.4)	2278 (45.6)
2024-2025	6950 (52.7)	1715 (39.3)	5235 (59.3)	2515 (65.6)	2720 (54.4)
Age					
Newborn (age <7 d)	8367 (63.4)	1949 (44.7)	6418 (72.7)	3832 (100)	2586 (51.7)
Nonnewborn (age ≥7 d), median (IQR), wk^a^	17.7 (8.1-26.9)	26.3 (11.1-28.4)	11.8 (4.9-20.6)	NA	11.8 (4.9-20.6)
Sex					
Female	6363 (48.2)	2040 (46.7)	4323 (49.0)	1890 (49.3)	2433 (48.7)
Male	6831 (51.8)	2325 (53.3)	4506 (51.0)	1941 (50.7)	2565 (51.3)
Unknown	1 (0.01)	0	1 (0.01)	1 (0.03)	0
Race and ethnicity					
Hispanic	1930 (14.6)	637 (14.6)	1293 (14.6)	462 (12.1)	831 (16.6)
Non-Hispanic Asian	2270 (17.2)	544 (12.5)	1726 (19.5)	798 (20.8)	928 (18.6)
Non-Hispanic Black	756 (5.7)	282 (6.5)	474 (5.4)	137 (3.6)	337 (6.7)
Non-Hispanic White	7085 (53.7)	2396 (54.9)	4689 (53.1)	2304 (60.1)	2385 (47.7)
Non-Hispanic other^b^	319 (2.4)	118 (2.7)	201 (2.3)	53 (1.4)	148 (3.0)
Unknown	835 (6.3)	388 (8.9)	447 (5.1)	78 (2.0)	369 (7.4)
Preterm (<37 wk)	1109 (8.4)	275 (6.6)	834 (9.6)	121 (3.2)	713 (14.3)
Unknown gestational age	282 (2.1)	166 (3.8)	116 (1.3)	1 (0.03)	115 (2.3)
Comorbidities (PMCA)					
No chronic disease	10 905 (82.6)	3620 (82.9)	7285 (82.5)	3368 (87.9)	3917 (78.4)
Non–complex chronic disease	1438 (10.9)	460 (10.5)	978 (11.1)	315 (8.2)	663 (13.3)
Complex chronic disease	852 (6.5)	285 (6.5)	567 (6.4)	149 (3.9)	418 (8.4)
Insurance status					
Public	1756 (13.3)	775 (18.3)	981 (11.3)	165 (4.3)	816 (16.6)
Private	11 208 (84.9)	3468 (79.5)	7740 (87.7)	3642 (95.0)	4098 (82.0)
Public plus private or unknown	231 (1.8)	122 (2.8)	109 (1.2)	25 (0.7)	84 (1.7)
Born at study institution	12 109 (91.8)	3777 (86.5)	8332 (94.4)	3832 (100)	4500 (90.0)
Clinical location					
Outpatient	5697 (43.2)	2035 (46.6)	3662 (41.5)	1434 (37.4)	2228 (44.6)
Newborn nursery	6078 (46.1)	1777 (40.7)	4301 (48.7)	2173 (56.7)	2128 (42.6)
NICU	612 (4.6)	96 (2.2)	516 (5.8)	89 (2.3)	427 (8.5)
Inpatient	250 (1.9)	176 (4.0)	74 (0.8)	54 (1.4)	20 (0.4)
Emergency department	363 (2.8)	281 (6.4)	82 (0.9)	82 (2.1)	0
Other^c^	195 (1.5)	0	195 (2.2)	0	195 (3.9)
Maternal language spoken					
English	12 549 (95.1)	4154 (95.7)	8395 (95.5)	3706 (96.9)	4689 (94.4)
Unknown	63 (0.5)	23 (0.5)	40 (0.5)	9 (0.2)	31 (0.6)
Maternal RSV vaccine status					
Received	4074 (30.9)	58 (1.3)	4016 (45.5)	3832 (100)	184 (3.7)
Not received	7664 (58.1)	3518 (80.6)	4146 (47.0)	NA	4146 (83.0)
Mother not documented	1457 (11.0)	789 (18.1)	668 (7.6)	NA	668 (13.4)
RSV immunization month^d^					
September	NA	NA	NA	472 (12.3)	NA
October	NA	NA	NA	713 (18.6)	1816 (36.3)
November	NA	NA	NA	795 (20.7)	990 (19.8)
December	NA	NA	NA	867 (22.6)	531 (10.6)
January	NA	NA	NA	949 (24.8)	359 (7.2)
February	NA	NA	NA	33 (0.9)	424 (8.5)
March	NA	NA	NA	2 (0.1)	878 (17.6)

Abbreviations: NA, not applicable; NICU, neonatal intensive care unit; PMCA, pediatric medical complexity algorithm; RSV, respiratory syncytial virus.

^a^ Study age defined as 0 for infants immunized through maternal RSV vaccine.

^b^ Includes American Indian or Alaska Native, Native Hawaiian or Other Pacific Islander, and other combinations not described.

^c^ Other locations refers to sites where location of nirsevimab receipt could not be determined.

^d^ Applies only to infants who had documented receipt of RSV immunization through maternal vaccination or nirsevimab.

### RSV Immunization in the 2023-2024 and 2024-2025 Seasons

**Figure 2.**
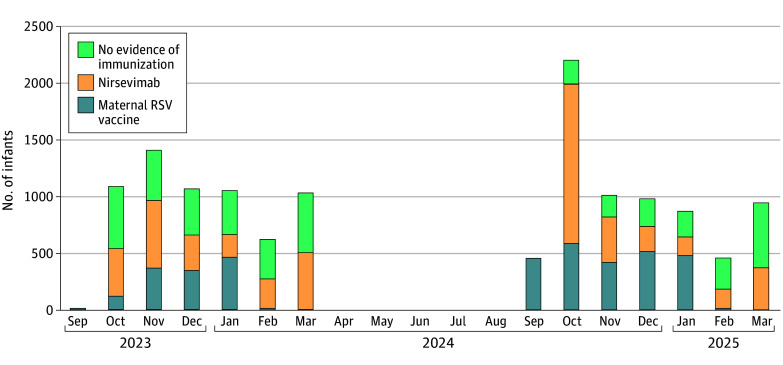
Monthly Administration of Maternal Respiratory Syncytial Virus (RSV) Vaccination or Nirsevimab Month for infants without evidence of immunization reflects the infants’ last clinical encounter in the RSV season.

During both RSV seasons, 8830 of 13 195 infants (66.9%) had evidence of RSV immunization from maternal RSV vaccine (3832 [29.0%]) or nirsevimab (4998 [37.9%]). In the 2023-2024 RSV season, 3595 of 6245 infants (57.6%) had evidence of RSV immunization: 1317 (21.1%) with maternal RSV vaccine and 2278 (36.5%) with nirsevimab. In the 2024-2025 season, 5235 of 6950 infants (75.3%) had evidence of RSV immunization: 2515 (36.2%) with maternal RSV and 2720 (39.1%) with nirsevimab, Of the 4365 infants without evidence of RSV immunization during the study, 789 (18.1%) had no documentation of maternal immunization status. Consistent with the seasonal rollout, nirsevimab administration was highest in October of the 2024-2025 season (Figure 2). We also evaluated receipt of maternal RSV vaccine and nirsevimab administered outside of the recommendations. Of 4365 infants without evidence of RSV immunization, 58 (1.3%) had been exposed to maternal RSV vaccine but not within the recommended 14 days or more prior to delivery. Of 4998 infants who received nirsevimab, 184 (3.7%) had also been exposed to maternal RSV vaccine at a median of 10 (IQR, 6-12) days prior to delivery. Forty-two of 3832 infants (1.1%) immunized through maternal RSV vaccine also received nirsevimab.

### Factors Associated With RSV Immunization

Factors associated with receiving maternal RSV vaccine or nirsevimab were evaluated in 12 693 infants with complete data available (Table 2). Infants seen in the 2024-2025 season had much greater odds of receiving maternal RSV vaccine (AOR, 3.58; 95% CI, 3.22-3.99) and almost double the odds of receiving nirsevimab (AOR, 1.89; 95% CI, 1.73-2.06) compared with the 2023-2024 season. Newborns had greater odds of receiving nirsevimab compared with older infants (AOR, 1.62; 95% CI, 1.48-1.78). Infants who had public insurance were much less likely to receive maternal RSV vaccine (AOR, 0.18; 95% CI, 0.15-0.22) and nirsevimab (AOR, 0.80; 95% CI, 0.70-0.89) compared with infants with private insurance, when controlling for RSV season, race and ethnicity, and age. Hispanic infants had slightly greater odds of receiving either maternal RSV vaccine or nirsevimab compared with non-Hispanic White infants (AOR for maternal RSV: 1.33 [95% CI, 1.12-1.57]; AOR for nirsevimab: 1.42 [95% CI, 1.25-1.63]), and non-Hispanic Asian infants also had higher odds of receiving either maternal RSV or nirsevimab compared to non-Hispanic White infants (AOR for maternal RSV, 1.71 [95% CI, 1.47-1.98]; AOR for nirsevimab. 1.74 [95% CI, 1.53-1.97]). Non-Hispanic Black infants had the same odds of receiving maternal RSV vaccine compared with non-Hispanic White infants; however, non-Hispanic Black infants were more likely to receive nirsevimab (AOR, 1.33; 95% CI, 1.10-1.61). Our findings remained unchanged across multiple sensitivity analyses (eTables 1-5 in Supplement 1). Additional analyses using nirsevimab as the reference category are provided in eTable 6 in Supplement 1.

**Table 2.** Multinomial Logistic Regression Analysis of Characteristics Associated With RSV Immunization Through Maternal RSV Vaccine or Nirsevimab

Characteristic	Outcome, AOR (95% CI)^a^	
	Maternal RSV vaccine	Nirsevimab
Age		
Nonnewborn	1 [Reference]	1 [Reference]
Newborn^b^	NA	1.62 (1.48-1.78)
RSV season		
2023-2024	1 [Reference]	1 [Reference]
2024-25	3.58 (3.22-3.99)	1.89 (1.73-2.06)
Gestational age		
Term (≥37 wk)	1 [Reference]	1 [Reference]
Prematurity (<37 wk)	0.70 (0.54-0.90)	2.66 (2.28-3.10)
Insurance		
Private insurance	1 [Reference]	1 [Reference]
Public insurance	0.18 (0.15-0.22)	0.80 (0.70-0.89)
Race and ethnicity		
Hispanic	1.33 (1.12-1.57)	1.42 (1.25-1.63)
Non-Hispanic Asian	1.71 (1.47-1.98)	1.74 (1.53-1.97)
Non-Hispanic Black	1.04 (0.80-1.36)	1.33 (1.10-1.61)
Non-Hispanic White	1 [Reference]	1 [Reference]

Abbreviations: AOR, adjusted odds ratio; RSV, respiratory syncytial virus.

^a^ Includes 12 693 infants with complete data for all variables.

^b^ Not estimated due to perfect collinearity between these factors.

### Location of Nirsevimab Receipt

The clinical location where nirsevimab was received varied by RSV season, with a significant difference in distribution between seasons (*P* < .001) (Table 3). During the 2023-2024 season, the most common site of nirsevimab receipt was in the newborn nursery (1271 of 2278 [55.8%]), followed by outpatient clinics (674 of 2278 [29.6%]). This trend shifted in the 2024-2025 season with outpatient clinics being the most common site (1554 of 2720 [57.1%]) of nirsevimab receipt, followed by the newborn nursery (857 of 2720 [31.5%]). In a secondary analysis including an interaction between clinical location and season, the observed shift in clinical location remained significant (eTable 7 in Supplement 1).

**Table 3.** Clinical Location of Nirsevimab Receipt by RSV Season

Clinical location	RSV season, No. (%)		*P* value^a^
	2023-2024 (n = 2278)	2024-2025 (n = 2720)	
Outpatient clinic	674 (29.6)	1554 (57.1)	<.001
Newborn nursery	1271 (55.8)	857 (31.5)	
Other			
NICU	236 (10.4)	191 (7.0)	
Inpatient	11 (0.5)	9 (0.3)	
Other	86 (3.8)	109 (4.0)	

Abbreviations: NICU, neonatal intensive care unit; RSV, respiratory syncytial virus.

^a^ Calculated using Pearson χ^2^ test comparing distribution of outpatient clinic, newborn nursery, and other locations.

## Discussion

Herein, we describe our experience of RSV immunization through exposure to maternal RSV vaccine or receipt of nirsevimab in a large cohort of infants who received medical care within a quaternary care hospital in New York during the first 2 RSV seasons these products were available. While coverage of RSV immunization improved during the 2024-2025 season, socioeconomic disparities persisted with either form of RSV immunization, particularly for infants who were publicly insured. Additionally, there was a shift in clinical locations where nirsevimab was received, with a lower proportion received in the newborn nursery during the 2024-2025 season, a finding which likely reflected increased availability in the outpatient settings.

We observed higher documented RSV immunization in the 2024-2025 compared with the 2023-2024 seasons, reflecting the implementation challenges of the first season. Our experience in the first RSV season was similar to what was reported nationally, where the rollout of 2 new vaccine products was hindered by the limited time between approval and period of eligibility (approximately 2-3 months), leaving minimal time to provide education and optimize operations and inconsistent availability across clinical locations.^9,10,11,12,13^ National rates of vaccine uptake were low, with only 29% of infants immunized against RSV through maternal vaccination (10.4%) or nirsevimab (18.5%), with state-wide rates ranging from 10.8% to 53.1%.^17^ The supply of nirsevimab was particularly limited in the 2023-2024 season, resulting in updated recommendations from the CDC prioritizing nirsevimab for infants younger than 6 months and for American Indian or Alaska Native children.^8^ Maternal RSV vaccine was also limited in certain clinical sites, including in our hospital, where the supply was delayed by a few weeks in Medicaid-insured clinics compared with privately insured sites, although in a prior report of pregnant individuals followed up at our institution,^12^ maternal RSV vaccine rates were 34.5%. Uptake of nirsevimab and maternal RSV during that season was largely dependent on the supply and consistent offering by practitioners, as institutions with ample supply reported high rates of uptake compared with what was being reported nationally.^9,11,12^ We suspect that limited supply was a major contributor to our rates in the 2023-2024 season, particularly in the early weeks of the season, when the manufacturer reported that the demand for nirsevimab had exceeded the supply.^8,18^

Several other factors not evaluated in this study may have contributed to lower rates of nirsevimab receipt during the initial rollout. As this was a new immunization product, some parents, especially those already hesitant about vaccines, may have been reluctant to accept nirsevimab. In a study of nirsevimab uptake at primary care sites in Pennsylvania,^14^ infants who had not received other vaccines had lower nirsevimab uptake than infants who were fully or partially vaccinated with other routine immunizations. Limited access to health care encounters may have also played a role, as lower nirsevimab uptake was observed among infants from neighborhoods with lower socioeconomic status.^14^ Variation in clinician prescribing practices and clinical workflow are additional potential factors.

It has been repeatedly demonstrated that vaccine uptake is associated with socioeconomic disparities.^19,20,21^ Prior reports of maternal RSV vaccine and nirsevimab uptake in the 2023-2024 RSV season found higher rates of uptake associated with private insurance and non-Hispanic ethnicity, and lower rates among those with public insurance and members of racial minority groups.^10,11,12,14,15^ In the present study, although rates of RSV immunization improved in the second season, with 75.3% of infants receiving either maternal RSV vaccine or nirsevimab, public insurance remained a significant barrier to equitable uptake, even when controlling for RSV season. On the other hand, unlike prior reports that found non-Hispanic Black race and Hispanic ethnicity to be associated with lower uptake, we found that Hispanic, non-Hispanic Asian, and non-Hispanic Black infants had slightly higher odds of RSV immunization compared with non-Hispanic White infants. Continued efforts to ensure equitable availability, offering, and education around the effectiveness and safety of maternal RSV vaccine and nirsevimab are essential to ensure equitable RSV protection to all infants.

Notably, the primary setting for nirsevimab administration shifted between the 2 RSV seasons, from predominantly in the newborn nursery in the 2023-2024 season to outpatient clinics in the 2024-2025 season. We suspect this change reflects the limited supply of nirsevimab in outpatient clinics in the 2023-2024 season and improved supply in the subsequent season. However, the magnitude of the decline in newborn nursery administration was unexpected, particularly in light of the CDC’s 2024-2025 recommendation to prioritize nirsevimab delivery at the earliest opportunity, including during the birth hospitalization.^22^ Early administration of infant vaccines is associated with improved uptake. Studies evaluating vaccine uptake for hepatitis B vaccine have found that administration in the nursery after birth was associated with higher rates of vaccine uptake and completion of the series.^23,24^ Determining the most effective setting for nirsevimab administration will be essential to achieving maximal reach across all populations of infants.

### Limitations

Our study has several limitations. First, our study was conducted at a single institution with a higher proportion of privately insured and English-speaking patients than many US populations, which may reflect higher access to prenatal counseling and fewer barriers to receiving the maternal vaccine or nirsevimab. Consequently, our RSV immunization rates may overestimate uptake in more diverse or resource-limited populations, limiting generalizability to other settings. However, the inclusion of multiple clinical settings within our institution enhances applicability across diverse health care environments. Second, as with all retrospective vaccine studies, our analysis depended on accurate documentation of vaccine administration. We could not definitively confirm that infants categorized as unimmunized did not receive maternal RSV vaccination or nirsevimab, as administration could have occurred outside our institution or geographic region. Nevertheless, documentation was robust, with 91% of infants born at our institution and nirsevimab receipt recorded in the Citywide Immunization Registry, which is integrated into the EHR. Despite a small proportion of infants excluded for missing vaccination data, our results were unchanged when including these infants in a sensitivity analysis. This limitation was more relevant to maternal RSV vaccination, as no citywide registry exists for maternal immunizations. Our institutional data indicate that 97.4% of individuals who deliver at our institution receive at least 1 prenatal care visit within our institution prior to delivery. While this does not eliminate the possibility of missing maternal vaccination data, as the prenatal visit may have not occurred within the vaccine eligibility period, this suggests a lower likelihood of misclassification of maternal vaccine status. Third, we could not assess other important factors associated with vaccine uptake, such as vaccine hesitancy, patient educational level, clinical workflow, and other patient-level barriers, as this was outside the scope of our study, but are paramount in understanding the full scope of immunization uptake.^25,26,27^ Fourth, we lacked data on nirsevimab supply and could not account for potential supply shortages during the 2023-2024 season. Last, we excluded infants at high risk older than 8 months of age who may have been eligible for nirsevimab, potentially underestimating overall uptake.

## Conclusions

In this large retrospective cohort study of infants eligible for RSV immunization through maternal vaccination or nirsevimab during the first 2 seasons of product availability, we observed significantly higher odds of receiving an RSV immunization product in the 2024-2025 season compared with the 2023-2024 season. Public insurance status was associated with lower uptake for both maternal RSV vaccination and nirsevimab, underscoring ongoing disparities in access despite overall gains in coverage. Future studies should aim to further our understanding of RSV immunization safety, effectiveness, and barriers to their uptake, particularly among underserved groups who are at higher risk for severe RSV disease and with limited access to RSV prevention. Moving forward, targeted strategies will be essential to ensure equitable access and optimize uptake across all sociodemographic groups.

## Notes

Supplement 1. **eMethods.** Sensitivity Analyses and Interaction Analysis of Clinical Location and Season **eTable 1.** Sensitivity Analysis With Excluded Patients (n = 635) Categorized as Immunized Through Maternal RSV Vaccine **eTable 2.** Sensitivity Analysis With Excluded Patients (n = 635) Categorized as Immunized Through Nirsevimab **eTable 3.** Sensitivity Analysis With Excluded Patients (n = 635) Categorized as No Evidence of Immunization **eTable 4.** Sensitivity Analysis Excluding Infants Who Had Received Both Maternal RSV Vaccine and Nirsevimab (n = 226) **eTable 5.** Sensitivity Analysis With Infants Who Received Maternal RSV Vaccine <14 Days Prior to Delivery (n = 58) Recategorized as Immunized Through Maternal RSV Vaccination **eTable 6.** Multinomial logistic Regression Model of Characteristics Associated With RSV Immunization Through Maternal RSV Vaccine or Nirsevimab, With Nirsevimab as Reference **eTable 7.** Parameter Estimates From Multinomial Logistic Regression Including the Interaction Terms Clinical Location With RSV Season

Supplement 2.
Data Sharing Statement
