# Supplementary material for: Infant Respiratory Syncytial Virus Immunization Through Maternal Vaccination and Nirsevimab
Source: JAMA Netw Open. 2026 Feb 16;9(2):e2559663. doi: 10.1001/jamanetworkopen.2025.59663 (PMC12910395; doi:10.1001/jamanetworkopen.2025.59663)
Supplement: Supplement 1. — eMethods. Sensitivity Analyses and Interaction Analysis of Clinical Location and Season eTable 1. Sensitivity Analysis With Excluded Patients (n = 635) Categorized as Immunized Through Maternal RSV Vaccine eTable 2. Sensitivity Analysis With Excluded Patients (n = 635) Categorized as Immunized Through Nirsevimab eTable 3. Sensitivity Analysis With Excluded Patients (n = 635) Categorized as No Evidence of Immunization eTable 4. Sensitivity Analysis Excluding Infants Who Had Received Both Maternal RSV Vaccine and Nirsevimab (n = 226) eTable 5. Sensitivity Analysis With Infants Who Received Maternal RSV Vaccine <14 Days Prior to Delivery (n = 58) Recategorized as Immunized Through Maternal RSV Vaccination eTable 6. Multinomial logistic Regression Model of Characteristics Associated With RSV Immunization Through Maternal RSV Vaccine or Nirsevimab, With Nirsevimab as Reference eTable 7. Parameter Estimates From Multinomial Logistic Regression Including the Interaction Terms Clinical Location With RSV Season [file jamanetwopen-e2559663-s001.pdf]

Supplementary Online Content

Acker KP, Strobino K, DeAngelis JM, et al. Infant respiratory syncytial virus immunization through maternal vaccination and nirsevimab. *JAMA Netw Open.* 2026;9(2):e2559663. doi:10.1001/jamanetworkopen.2025.59663

eMethods. Sensitivity Analyses and Interaction Analysis of Clinical Location and Season

eTable 1. Sensitivity Analysis With Excluded Patients (n = 635) Categorized as Immunized Through Maternal RSV Vaccine

eTable 2. Sensitivity Analysis With Excluded Patients (n = 635) Categorized as Immunized Through Nirsevimab

eTable 3. Sensitivity Analysis With Excluded Patients (n = 635) Categorized as No Evidence of Immunization

eTable 4. Sensitivity Analysis Excluding Infants Who Had Received Both Maternal RSV Vaccine and Nirsevimab (n = 226)

eTable 5. Sensitivity Analysis With Infants Who Received Maternal RSV Vaccine <14 Days Prior to Delivery (n = 58) Recategorized as Immunized Through Maternal RSV Vaccination

eTable 6. Multinomial logistic Regression Model of Characteristics Associated With RSV Immunization Through Maternal RSV Vaccine or Nirsevimab, With Nirsevimab as Reference

eTable 7. Parameter Estimates From Multinomial Logistic Regression Including the Interaction Terms Clinical Location With RSV Season

This supplementary material has been provided by the authors to give readers additional information about their work.

© 2026 Acker KP et al. *JAMA Network Open*.

eMethods. Sensitivity Analyses and Interaction Analysis of Clinical Location and Season

Sensitivity analyses.

We performed multiple sensitivity analyses to evaluate the robustness of our findings. First, to account for infants with unavailable vaccination records, a sensitivity analysis was performed to assess how the multinomial regression model was affected if excluded infants were categorized in each outcome strata (i.e., maternal RSV vaccine, nirsevimab, not immunized) (**eTable1-3**). Second, we excluded infants who had received both maternal RSV vaccine and nirsevimab (**eTable4**). Third, we re-categorized infants who had received maternal RSV vaccine within 14 days prior to delivery (and thus outside of the recommended time-frame of receipt) as immunized by maternal RSV vaccine (**eTable5**).

Interaction analysis of clinical location and season

A secondary analysis was performed to assess whether seasonal differences in the clinical location of nirsevimab administration persisted after adjustment for patient characteristics. Clinical location (newborn nursery vs other locations) was included in the multinomial regression model as an interaction term with RSV season (2023-2024 vs 2024-2025), using the same covariates as in the primary adjusted model (**eTable7**).

© 2026 Acker KP et al. *JAMA Network Open*.

eTable 1. Sensitivity Analysis With Excluded Patients (n = 635) Categorized as Immunized Through Maternal RSV Vaccine

Characteristic		Outcome	
		**Maternal RSV vaccine** Adjusted OR (95% CI)	**Nirsevimab** Adjusted OR (95% CI)
Age	Non-newborn	Reference	Reference
	Newborn[a]	NA	1.62 (1.48-1.78)
RSV season	2023-2024	Reference	Reference
	2024-25	3.21 (2.91-3.55)	1.89 (1.73-2.05)
Gestational age	Term (≥37 weeks)	Reference	Reference
	Prematurity (<37 wks)	1.06 (0.85-1.32)	2.66 (2.29-3.10)
Insurance	Private insurance	Reference	Reference
	Public insurance	0.36 (0.31-0.43)	0.82 (0.73-0.93)
Race/ethnicity	Non-Hispanic White<br>Hispanic<br>Non-Hispanic Asian<br>Non-Hispanic Black	Reference<br>1.25 (1.07-1.45)<br>1.60 (1.39-1.84)<br>1.06 (0.84-1.35)	Reference<br>1.40 (1.23-1.60)<br>1.74 (1.53-1.98)<br>1.32 (1.09-1.59)

[a]Odds ratio for newborn status on maternal RSV vaccine receipt not estimated due to perfect collinearity between these factors.

© 2026 Acker KP et al. *JAMA Network Open*.

eTable 2. Sensitivity Analysis With Excluded Patients (n = 635) Categorized as Immunized Through Nirsevimab

Characteristic		Outcome	
		**Maternal RSV vaccine** Adjusted OR (95% CI)	**Nirsevimab** Adjusted OR (95% CI)
Age	Non-newborn	Reference	Reference
	Newborn[a]	NA	1.61 (1.47-1.75)
RSV season	2023-2024	Reference	Reference
	2024-25	3.55 (3.19-3.95)	1.92 (1.76-2.08)
Gestational age	Term (≥37 weeks)	Reference	Reference
	Prematurity (<37 wks)	0.65 (0.50-0.83)	2.76 (2.38-3.21)
Insurance	Private insurance	Reference	Reference
	Public insurance	0.19 (0.16-0.24)	0.87 (0.78-0.98)
Race/ethnicity	Non-Hispanic White	Reference	Reference
	Hispanic	1.30 (1.10-1.54)	1.35 (1.19-1.54)
	Non-Hispanic Asian	1.69 (1.46-1.95)	1.67 (1.48-1.89)
	Non-Hispanic Black	1.03 (0.79-1.34)	1.30 (1.08-1.56)

[a]Odds ratio for newborn status on maternal RSV vaccine receipt not estimated due to perfect collinearity between these factors.

© 2026 Acker KP et al. *JAMA Network Open*.

eTable 3. Sensitivity Analysis With Excluded Patients (n = 635) Categorized as No Evidence of Immunization

Characteristic		Outcome	
		**Maternal RSV vaccine** Adjusted OR (95% CI)	**Nirsevimab** Adjusted OR (95% CI)
Age	Non-newborn	Reference	Reference
	Newborn[a]	NA	1.59 (1.45-1.73)
RSV season	2023-2024	Reference	Reference
	2024-25	3.38 (3.05-3.76)	1.79 (1.65-1.95)
Gestational age	Term (≥37 weeks)	Reference	Reference
	Prematurity (<37 wks)	0.53 (0.42-0.67)	2.17 (1.88-2.50)
Insurance	Private insurance	Reference	Reference
	Public insurance	0.18 (0.15-0.22)	0.75 (0.67-0.85)
Race/ethnicity	Non-Hispanic White	Reference	Reference
	Hispanic	1.34 (1.13-1.58)	1.43 (1.26-1.63)
	Non-Hispanic Asian	1.73 (1.50-2.01)	1.73 (1.57-2.01)
	Non-Hispanic Black	1.03 (0.80-1.34)	1.32 (1.10-1.58)

[a]Odds ratio for newborn status on maternal RSV vaccine receipt not estimated due to perfect collinearity between these factors.

© 2026 Acker KP et al. *JAMA Network Open*.

eTable 4. Sensitivity Analysis Excluding Infants Who Had Received Both Maternal RSV Vaccine and Nirsevimab (n = 226)

Characteristic		Outcome	
		**Maternal RSV vaccine** Adjusted OR (95% CI)	**Nirsevimab** Adjusted OR (95% CI)
Age	Non-newborn	Reference	Reference
	Newborn[a]	NA	1.57 (1.43-1.72)
RSV season	2023-2024	Reference	Reference
	2024-25	3.65 (3.27-4.06)	1.86 (1.70-2.03)
Gestational age	Term (≥37 weeks)	Reference	Reference
	Prematurity (<37 wks)	0.70 (0.54-0.90)	2.41 (2.06-2.81)
Insurance	Private insurance	Reference	Reference
	Public insurance	0.16 (0.13-0.20)	0.81 (0.72-0.92)
Race/ethnicity	Non-Hispanic White	Reference	Reference
	Hispanic	1.34 (1.13-1.59)	1.42 (1.25-1.63)
	Non-Hispanic Asian	1.73 (1.50-2.00)	1.73 (1.53-1.97)
	Non-Hispanic Black	1.05 (0.80-1.37)	1.32 (1.10-1.60)

[a]Odds ratio for newborn status on maternal RSV vaccine receipt not estimated due to perfect collinearity between these factors.

© 2026 Acker KP et al. *JAMA Network Open*.

eTable 5. Sensitivity Analysis With Infants Who Received Maternal RSV Vaccine <14 Days Prior to Delivery (n = 58) Recategorized as Immunized Through Maternal RSV Vaccination

Characteristic		Outcome	
		**Maternal RSV vaccine** Adjusted OR (95% CI)	**Nirsevimab** Adjusted OR (95% CI)
Age	Non-newborn	Reference	Reference
	Newborn[a]	NA	1.65 (1.50-1.80)
RSV season	2023-2024	Reference	Reference
	2024-25	3.65 (3.27-4.06)	1.90 (1.74-2.08)
Gestational age	Term (≥37 weeks)	Reference	Reference
	Prematurity (<37 wks)	0.70 (0.54-0.90)	2.80 (2.40-3.27)
Insurance	Private insurance	Reference	Reference
	Public insurance	0.18 (0.15-0.22)	0.77 (0.68-0.88)
Race/ethnicity	Non-Hispanic White	Reference	Reference
	Hispanic	1.33 (1.13-1.57)	1.43 (1.25-1.64)
	Non-Hispanic Asian	1.74 (1.50-2.02)	1.77(1.56-2.01)
	Non-Hispanic Black	1.13 (0.87-1.47)	1.37 (1.13-1.66)

[a]Odds ratio for newborn status on maternal RSV vaccine receipt not estimated due to perfect collinearity between these factors.

© 2026 Acker KP et al. *JAMA Network Open*.

eTable 6. Multinomial logistic Regression Model of Characteristics Associated With RSV Immunization Through Maternal RSV Vaccine or Nirsevimab, With Nirsevimab as Reference

Characteristic		**Maternal RSV vaccine**<br>**Ref = Nirsevimab**<br>Adjusted OR (95% CI)
Age	Non-newborn	Reference
	Newborn[a]	NA
RSV season	2023-2024	Reference
	2024-25	1.90 (1.71-2.10)
Gestational age	Term (≥37 weeks)	Reference
	Prematurity (<37 wks)	0.26 (0.21-0.33)
Insurance	Private insurance	Reference
	Public insurance	0.23 (0.19-0.28)
Race/ethnicity	Non-Hispanic White<br>Hispanic<br>Non-Hispanic Asian<br>Non-Hispanic Black	Reference<br>0.93 (0.80, 1.09)<br>0.98 (0.86, 1.12)<br>0.79 (0.61, 1.01)

[a]Odds ratio for newborn status on maternal RSV vaccine receipt not estimated due to perfect collinearity between these factors.

When using nirsevimab as the reference category in our model, infants with public insurance demonstrated lower odds of receiving maternal RSV vaccine (aOR 0.23, 95% CI 0.19-0.28). Infants seen during the 2024-2025 season had slightly higher odds of receiving maternal RSV vaccine (aOR 1.90, 95% CI 1.71-2.10) compared to nirsevimab.

© 2026 Acker KP et al. *JAMA Network Open*.

eTable 7. Parameter Estimates From Multinomial Logistic Regression Including the Interaction Terms Clinical Location With RSV Season

Effect	DF	Wald Chi-Square	p-value
Newborn status	2	445.42	<0.0001
Race/ethnicity	8	138.69	<0.0001
Preterm status	2	292.60	<0.0001
Insurance type	2	282.37	<0.0001
Indicator for maternal RSV vaccine recommendation period (dummy variable)[a]	2	206.73	<0.0001
Season * clinical location interaction	4	877.39	<0.0001

[a]This variable indicated whether the infant was seen at a time when maternal RSV vaccination was recommended (September to January), or not recommended (February to March).
