# Supplementary material for: Infant Respiratory Syncytial Virus Immunization Through Maternal Vaccination and Nirsevimab
Source: JAMA Netw Open. 2026 Feb 16;9(2):e2559663. doi: 10.1001/jamanetworkopen.2025.59663 (PMC12910395; doi:10.1001/jamanetworkopen.2025.59663)
Supplement: Supplement 2. — Data Sharing Statement [file jamanetwopen-e2559663-s002.pdf]

Data Sharing Statement

Acker. Infant Respiratory Syncytial Virus Immunization Through Maternal Vaccination and Nirsevimab. *JAMA Netw Open*. Published February 16, 2026.
doi:10.1001/jamanetworkopen.2025.59663

Data
Data available: No

Additional Information
Explanation for why data not available: Data will be made available upon reasonable request.
